# Toxicity mechanism of lead on bumblebees: from gene expression to gut microbiota analysis

**DOI:** 10.3389/fmicb.2026.1821968

**Published:** 2026-06-29

**Authors:** Wang Shuang, Meng Qingxin, Xue Yunfei, Yue Dan, Tian Weixue, Huang Rong, Guo Shuai, Zhou Yu, Tian Yakai, Cao Zhenhui, Dong Kun

**Affiliations:** Yunnan Provincial Engineering and Research Center for Sustainable Utilization of Honey Bee Resources, Eastern Bee Research Institute, College of Animal Science and Technology, Yunnan Agricultural University, Kunming, China

**Keywords:** bumblebees, gene expression, gut microbiota, heavy metal pollution, lead

## Abstract

**Introduction:**

Heavy metal pollution poses a persistent global threat to pollinator health and biodiversity. Lead is environmentally stable and can induce multifaceted physiological disorders in pollinating insects. However, the comprehensive toxic impacts of lead on bumblebees, particularly the interconnections between host gene expression and gut microbiota, remain largely underexplored.

**Methods:**

In this study, *Bombus terrestris* individuals were chronically exposed to three lead concentrations (0.95, 3.6, and 80 mg/L), selected based on acute toxicity data and field-relevant pollution levels. Following oral exposure via sugar water, we examined the expression profiles of genes associated with learning and memory, detoxification, and immune defense, as well as structural alterations in gut microbial communities.

**Results:**

Lead exposure significantly altered the transcription of *DopR1*, *DopR2*, *NMDA*, *GST*, *PPO*, *defensin*, and *Hymen*. Gut microbiota profiling further revealed distinct operational taxonomic unit (OTU) distributions across treatment groups, indicating that lead exposure reshaped the diversity and community structure of bumblebee gut microbes.

**Discussion:**

These findings uncover the coordinated toxic responses of host genes and gut bacteria under lead stress, and provide critical insights for a more comprehensive assessment of the ecological risks that heavy metal pollution poses to bumblebee populations.

## 1 Introduction

Pollinating insects play a crucial role in ensuring food security and preserving biodiversity by providing essential pollination services to both natural ecosystems and agricultural crop (Garibaldi et al., 2013). Among these, bumblebees are particularly important for plant reproduction due to their high pollination efficiency and strong adaptability to environmental changes. As such, they are often used as supplementary pollinators, especially when honeybee populations decline or nectar sources are limited (Timberlake et al., 2019).

Moreover, bumblebees’ sensitivity to environmental changes makes them valuable biological indicators for assessing ecosystem health (Weis et al., 2017).

In recent years, rapid industrialization has improved living standards but also exacerbated environmental pollution, particularly from heavy metals (Ahmed et al., 2022). Indeed, these metals enter ecosystems through both natural processes and anthropogenic activities, and since they are non-degradable as well as potentially toxic, they tend to accumulate and persist in the environment (Ahmed et al., 2023). Notably, when heavy metals infiltrate the nectar and pollen of flowering plants, pollinator visitation rates may decline (Xun et al., 2018). This is because pollinators can become contaminated either by heavy metal-laden pollen adhering to their body hairs during foraging or by inhaling airborne metal particles through their spiracles, leading to adverse effects on their health (Benner et al., 2025).

Lead, a persistent and widely used heavy metal found in chemicals, cables, batteries, and radiation shielding, is particularly concerning (Rahman and Singh, 2019). In fact, this metal damages cell structure, inhibits enzyme activity, and interferes with DNA transcription and neurotransmitter function in insects, thereby impairing cell metabolism, immune responses, and genetic integration (Bashir et al., 2016). Previous studies have also shown that lead exposure increases pupal mortality, alters learning and cognitive abilities, and accumulates in bumblebees over time (Filipiak et al., 2017; Burden et al., 2019). In fact, it can also be detected in hives following prolonged exposure (Schmarsow et al., 2023).

Insect intestines contain a variety of microorganisms that perform essential functions for their hosts, such as nutrient acquisition and food digestion. These microorganisms also influence the growth, development, and reproduction of their hosts (Mannaa et al., 2024). The composition of the intestinal microbiota can be altered by environmental factors. Bumblebees (genus Bombus) and Western honeybees (*Apis mellifera)* represent classic model systems for investigating insect gut microbiota (Kwong et al., 2014). Phylogenetically closely related, these pollinators possess gut microbial communities of profound physiological and ecological importance. Previous work has consistently documented that they carry a specialized, low-diversity gut microbiota. This microbiota is dominated by certain core bacteria, including *Gilliamella* (*Gamma-1 strain*; *Gammaproteobacteria*; C*oscinodictyaceae*),(*Gammaproteobacteria*; K*nitinectaceae*), *Lactobacillus* spp.(*Firm-4*/*Lacto-2* and *Firm-5*/*Lacto-1*; *Firmicutes*; *Lactobacillaceae*) and *Bifidobacterium* spp. (Martinson et al., 2011; Koch and Schmid-Hempel, 2011; Meeus et al., 2015). However, the microbiota of pollinating insects can be adversely affected by exposure to heavy-metal pollution during food collection. The disruptive actions of heavy metals interfere with the beneficial interactions between intestinal microorganisms and their hosts, thereby reducing the adaptability and resistance of the host (Li et al., 2024). Therefore, investigation of the mechanisms by which a heavy metal such as lead affects the intestinal microbiota of bumblebees is important for the conservation and promotion of bumblebee breeding.

However, the toxic effects of lead exposure on bumblebees most likely do not depend on a single mechanism. On one hand, lead exposure may lead directly to changes in the expression of genes related to intestinal detoxification and immunity in bumblebees, thereby selecting lead-tolerant microbial populations and consequent alterations in the composition and function of the gut microbiota. On the other hand, changes in the microbiota composition may, via altering metabolite levels and nutrient absorption, negatively affect the expression of host genes, thereby influencing both the detoxification efficiency and tolerance of bumblebees to lead. Therefore, the purpose of this study was to monitor mortality rates in bumblebees exposed to sublethal doses of lead to analyze changes in the expression of genes related to learning and memory, as well as genes related to intestinal detoxification, oxidative stress, and immunity. The study also investigated changes in the composition of the gut microbiota following long-term exposure to three different concentrations of lead. The findings provide new insights into the mechanisms underlying heavy-metal toxicity in pollinating insects and offer important clues for understanding the broader ecological impacts of heavy-metal pollution on these organisms.

## 2 Materials and methods

### 2.1 Bumblebee rearing

In October 2024, 10 early-stage colonies of *Bombus terrestris* were purchased from Yunnan Biote (Shou guang) Biotechnology Co., Ltd. (China). Bumblebee colonies were housed in a dark laboratory maintained at 29 ± 1 °C with 55% relative humidity. Newly emerged individuals were collected under red light and placed into pre-treated plastic boxes for acclimation and subsequent feeding. Newly emerged workers were selected under red light and transferred to pre-treated plastic boxes for acclimatization and feeding. Bees were provided with 50% (w/v) sucrose solution delivered via preassembled syringes. Before use, each syringe was carefully checked for leaks to ensure consistent feeding volume.

### 2.2 Preparation of lead-containing compounds

Lead(II) chloride (PbCl₂; CAS: 7758-95-4; molecular weight: 278.11; purity ≥ 99.5%) was purchased from Shanghai Macklin Biochemical Technology Co., Ltd. (China). A stock solution of PbCl₂ was prepared in 50% (w/v) sucrose solution and stored at 4 °C until use. All dilutions for experimental feeding were freshly prepared before each exposure period. A three-channel real-time fluorescence quantitative PCR instrument (CFX Connect Real-Time System, manufacturer: Bio-Rad Laboratories, model: CFX Connect Real-Time System) was used for subsequent qPCR analyses.

### 2.3 Acute and chronic toxicity experiments

Acute toxicity experiment: Adult worker bees of similar size were randomly selected from the 10 colonies and placed into cages for feeding. Six lead concentration gradients (Pb groups: 1000, 2,500, 4,000, 5,500, 7,000 and 8,000 mg/L) were prepared using 50% sucrose solution, with a control group receiving pure 50% sucrose solution also included. In this case, each experimental group included three replicates, with 24 Bumblebee per cage. All feeding solutions were stored at 4 °C and replaced every 24 h. Bees were kept in a dark rearing room (29 ± 1 °C, 55% RH), with mortality recorded after 72 h of exposure. Feeding syrups were sterilized at high temperature before use to ensure microbial safety. The median lethal concentration (LC_50_) of lead was eventually calculated using Probit analysis in SPSS 26.0software.

Lead concentrations between 0.08 and 3.6 mg/kg have been reported in bumblebees (Breidenbach et al., 2023). Therefore, the present study used 3.6 mg/L as an intermediate lead concentration for bumblebee exposure. Additionally, considering that the highest lead concentration detected in Bumblebee was 0.89 mg/kg (Gutierrez et al., 2020), we set a lower limit of 0.95 mg/L, which covers the highest lead concentration ever observed in bees. Furthermore, based on the strictest allowable limit for lead concentrations in soil, namely, 80 mg/L, we applied this value as the highest concentration for the lead-exposure experimental group (Sivakoff and Gardiner, 2017); this value represents the concentration of lead that is present in the environment but inaccessible to bumblebees. In addition, a control group (CK) that received only a 50% sucrose solution was included. Each treatment group had six replicates, and the bees were maintained under the same environmental conditions as in the acute test, with feeding solutions replaced daily. Finally, mortality and feed intake were recorded throughout the 15-day exposure period.

### 2.4 Sample preparation

After 15 days of chronic exposure, the surviving bees from each group (L, M, H, and CK) were anesthetized by freezing. Their heads and abdomens were then dissected, collected into 1.5-mL centrifuge tubes, and homogenized to create pooled samples of head and abdominal tissue. In this study, each biological replicate consisted of five bumblebees, resulting in a total of six replicates per group. The low yield of RNA from individual bumblebee head/abdominal tissues (approximately 5–10 ng per individual) made it impossible to meet the minimum quality requirements for cDNA synthesis and qPCR analysis (≥30 ng). Additionally, this approach was selected for biological reasons, as it contributed to reduced variation between individual specimens while maintaining the statistical validity of the results. The use of six replicates per group compensated for the effects of using combined data on the sample size, thereby ensuring the reliability of the statistical analyses. These samples were ultimately used to analyze the expression of gene related to memory, detoxification, and immunity. All samples were stored at −80 °C in ultra-low temperature freezers.

### 2.5 Gene expression analysis

Total RNA, extracted from the head and gut samples, was reverse-transcribed into cDNA using a commercial reverse transcription kit. qPCR (the reagents and dosages used are listed in Table 1) (YEASEN)was then conducted under the following conditions: pre-denaturation at 94 °C for 30 s, followed by 40 cycles, each consisting of denaturation at 94 °C for 5 s and annealing at 60 °C for 34 s. Relative gene expression levels were subsequently calculated using the 2^-ΔΔCt^ method, The internal reference gene was *β-actin*, and the values of M ranged from 0.28 to 0.45, all of which were below the stable threshold of 0.5. Additionally, the paired coefficient of variation V2/3 was less than 0.15, meeting the core requirements for using a gene as an internal reference in qPCR analyses (Sankar et al., 2022). In this experiment, the head samples were analyzed for associative memory-related genes (*DopR1*, *DopR2*, *NMDA*), while the gut samples were tested for genes related to detoxification (*GST*) and immune response (*PPO*, *defensin*, *Hymen*). Details of the primers used are provided in Table 2.

**Table 1:** qPCR reaction system.

Reagent name	20 μL system	Final concentration
2xqPCR SYBR Green Master Mix (No Rox)	10 μL	1×
Forward Primer (10um)	0.4 μL	0.2um
Reverse Primer (10um)	0.4 μL	0.2um
Template DNA	3.2 μL	1–10 ng
RNase-Free ddH_2_O	6 μL	–

**Table 2:** Primer sequences used for the amplification of selected genes.

Gene	Primer sequences(5′-3′)
*DopR1*	F: CCCGTAATGTATGATGATGGTAAAG R: CGATGCAGGGCACGTAAAA
*DopR2*	F: GGAGGAAGTGCCAGAGGACA R: TCACGAACAGGGGTAAGTAGAAAC
*NMDA*	F: GAGTCTTCATAGTGGTCGGTGTTG R: TGTCTCGCCAGTTCCATCTTT
*GST*	F: GAAGATAAGAGGCTCACTCAGGAT R: GTCGATTTCCAAGACGGGTAC
*PPO*	F: TGCTTGCTAGATCCACTCACTTG R: GGACCTATGAAGATGCGAACAG
*defensin*	F: TGCCGATAGACAAAGAAGAGTGA R: TTTGCCCATGCTGAGACAGT
*Hymen*	F: GCTGCCAGAATTGAACCTGA R: CCGCTCAATGGTTTCTTTCC
*β-actin*	F: CGACTACCTCATGAAGATT R: CGACGTAACAAAGTTTCTC

### 2.6 Gut microbiota analysis of bumblebees

The hindguts of surviving bumblebees were dissected for gut microbiota analysis, which was conducted by Majorbio (Shanghai, China). Specifically, full-length 16S rRNA genes were amplified using the primers 27F (5’-AGRGTTYGATYMTGGCTCAG-3′) and 1492R [5’-RGYTACCTTGTTACGACTT-3′. This technology is characterized by extremely long read lengths (average read length > 10 kb), enabling complete coverage of the 16S rRNA gene sequence (V1-V10 regions, approximately 1,500 bp)] (Klindworth et al., 2013). Using the CCS (Circular Consensus Sequencing) mode of SMRT Link v11.0 software, high-fidelity full-length sequences were generated from the raw subreads, with a sequence accuracy of 99.9%. Sample data were demultiplexed based on barcode sequences, and only bacterial 16S rRNA gene sequences of 1,000–1800 bp length were retained. Abnormal sequences shorter than 1,000 bp or longer than 1800 bp were removed. UPARSE version 7.1 software was used for clustering based on a 97% sequence similarity threshold. UCHIME algorithm was applied to remove potential chimeric sequences. Sequences annotated as belonging to chloroplasts or mitochondria were manually filtered out to avoid interference from non-target microorganisms. To minimize the impact of varying sequencing depths on diversity analysis, the number of sequences per sample was adjusted to 6,000. Even after adjustment, the average sequence coverage of each sample remained at 99.09%, ensuring reliable analysis results. Taxonomic assignment of OTUs was performed using RDP Classifier version 2.11 and the Silva 16S rRNA gene database (v138). Annotation levels included phylum, class, order, family, genus, and species. The confidence threshold was set at 0.7. Alpha diversity indices (Chao, Shannon, Simpson, ACE) were calculated using mothur software. Beta diversity analysis was performed using PCoA based on Bray-Curtis distances, with PERMANOVA non-parametric tests to assess significance of differences in community structure between groups. LEfSe analysis was used to identify species with significant differences between groups (LDA > 2.0, *p* < 0.05).

### 2.7 Statistical analysis

All data were first presented as mean ± standard error (SE). Their normality was assessed using the Kolmogorov–Smirnov test, with the results confirming that all datasets followed a normal distribution. In addition, homogeneity of variances was determined using the Brown-Forsythe test: a *p*-value < 0.05 indicated heterogeneous variances (violation of the homogeneity assumption), while *p* > 0.05 indicated homogeneous variances. For inter-group comparisons, one-way ANOVA followed by Tukey’s *post hoc* test was performed in SPSS 26.0 when variances were homogeneous (*p* > 0.05). When variances were heterogeneous *(p* < 0.05), non-parametric tests (Kruskal-Wallis H test followed by Dunn’s post hoc test) were employed to avoid misleading results. Finally, survival curve differences among groups were analyzed using the Log-rank test (Mantel-Cox) in GraphPad Prism 10.3. The levels of significance were *p < 0.05, **p < 0.01, ***p < 0.001, and *p* ≥ 0.05 (non-significant). For pairwise difference grading, different lowercase letters (a, b, c) indicate significant differences among groups (*p* < 0.05), while the same letter indicates no significant difference (*p* ≥ 0.05).

## 3 Results

### 3.1 Lead toxicity in *Bombus terrestris*

As shown in Table 3, when *Bombus terrestris* was exposed to lead concentration for 72 h, the calculated LC_50_ was 3945.3 mg a.i L^−1^, (F. L95% = −251.017—5307.57 mg a.i. L-1; *χ*^2^ = 6.472). The median lethal concentration (LC₅₀) of lead was calculated using Probit regression analysis. The goodness-of-fit of the Probit model was evaluated using the Pearson chi-square (χ^2^) test, with *p* > 0.05 indicating an acceptable fit.

**Table 3:** Toxicity of lead to bumblebees (*Bombus terrestris).*

Exposure time (h)	pb			
	df	*χ* ^2^	*p*	LC_50_ (95% FL) mg a.i. L-1
72 h	4	0.167	6.472	3945.3(−251.017---5307.57)

LC₅₀: median lethal concentration at 72 h; *χ*^2^: Pearson chi-square goodness-of-fit value; df: degrees of freedom; *p* > 0.05 indicates the Probit model is well fitted.

### 3.2 Effects of lead on bumblebee survival and food intake

**Figure 1:**
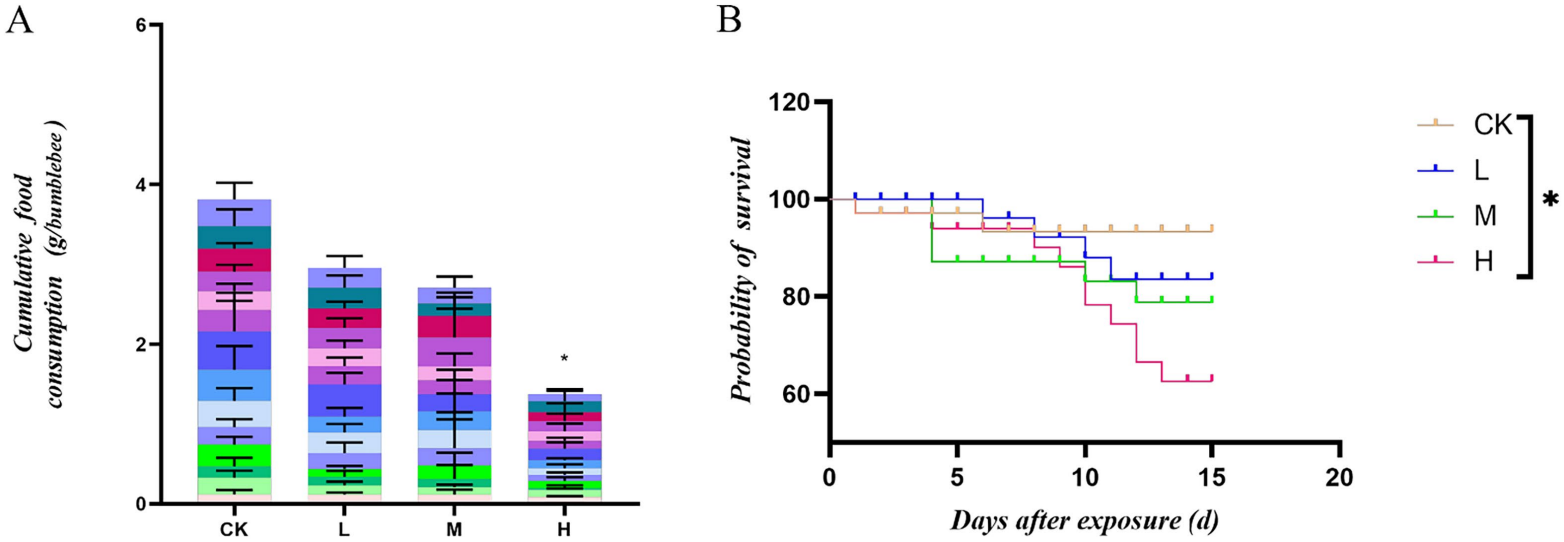
Effects of sub-lethal lead exposure on bumblebee survival and food intake over 15 days. **(A)** Survival rates of CK, L, M, and H groups. **(B)** Cumulative food intake of each group. Data are presented as mean ± SE. *p < 0.05.

there was a progressive decline in food consumption in the lead-exposed group (L, M and H) as the exposure time increased. Notably, food intake in the H group was significantly lower than that in the control group, suggesting that lead exposure negatively affected feeding behavior (Figure 1A). In particular, the survival rate of bumblebees exposed to different sublethal concentrations of lead over a 15-day period is shown in Figure 1B. Overall, the mortality increased with lead concentration, indicating a dose-dependent effect. The high-dose lead group (H) exhibited a significantly higher mortality rate compared with the control group (CK) (*p* < 0.05).

### 3.3 Expression of memory-related genes in bumblebee heads

**Figure 2:**
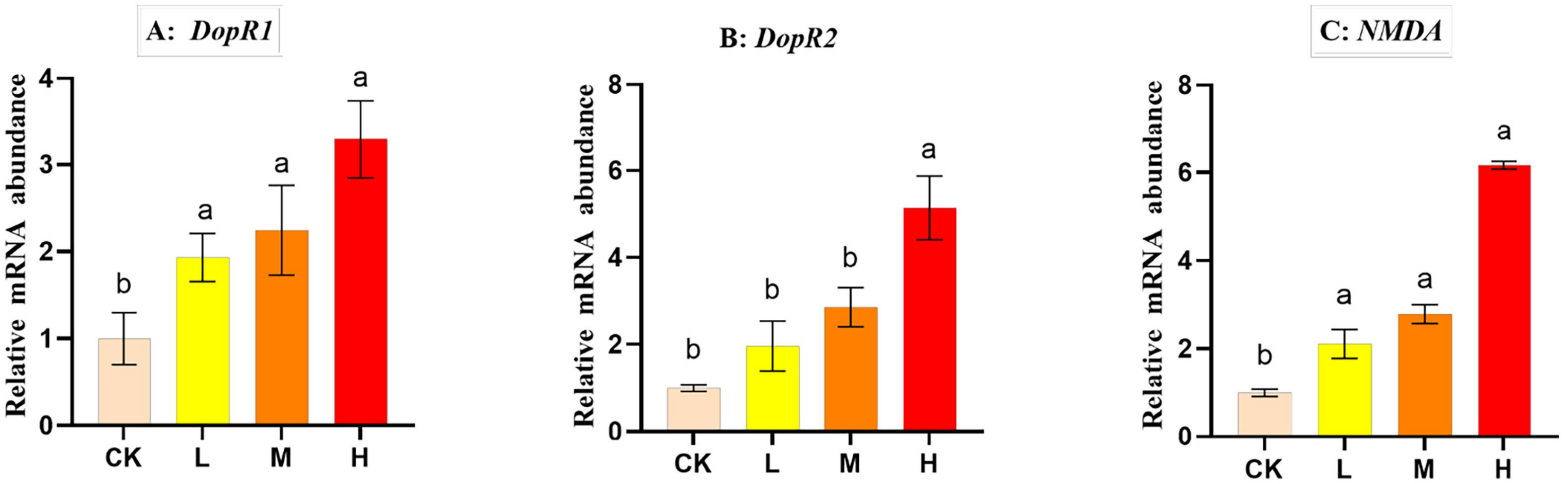
Expression levels of memory-related genes: **(A)** *DopR1,* **(B)** *DopR2,* and **(C)** *NMDA* in bumblebee heads after 15 days of lead exposure. Different letters (a and b) indicate significant differences compared with the control group (*p* < 0.05); relative expression (2^-ΔΔCt^) normalized to CK; CK = control group (50% sucrose solution); L, M, and H represent the low, medium, and high lead concentrations, respectively.

The genes *DopR1, DopR2*, and *NMDA* are associated with learning and memory in insects (Zhu et al., 2020). In this study, the relative expression of *DopR1* increased with lead concentration, with the H group showing significantly higher expression than the CK group (Figure 2A) (*p* < 0.05). Similarly, *DopR2* expression peaked in the H group, which was significantly higher than in the CK, L and M groups (Figure 2B) (*p* < 0.05). Finally, *NMDA* gene expression was also significantly elevated in all lead-treated groups (L, M and H) compared with CK (*p* < 0.05), with the highest expression observed in the H group (Figure 2C).

### 3.4 Effects of lead on bumblebee gut immunity

**Figure 3:**
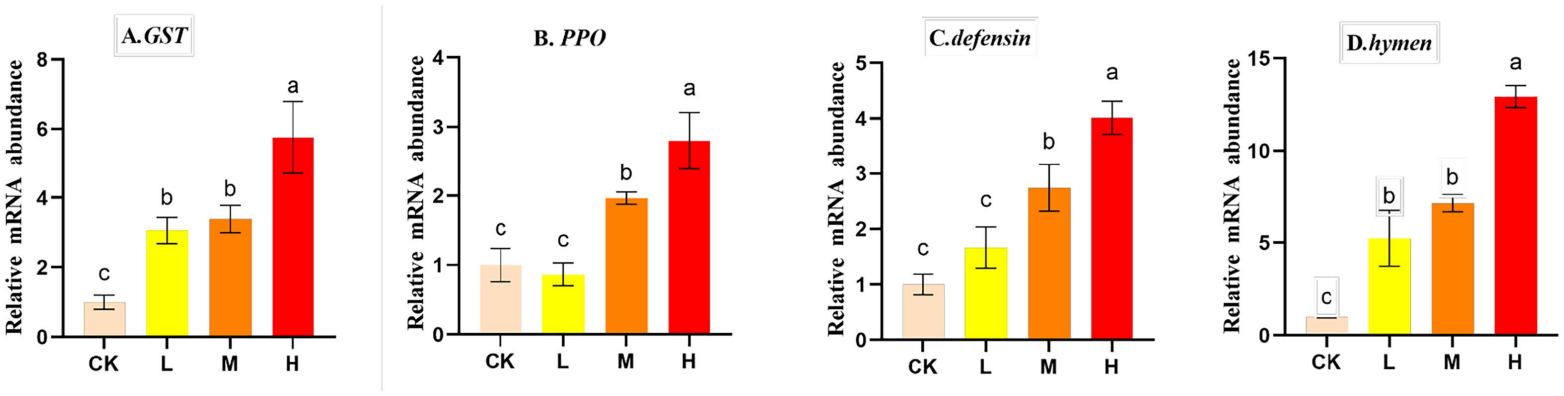
Expression of gut detoxification and immunity-related genes after 15 days of lead exposure. **(A)** *GST*; **(B)** *PPO*; **(C)** *defensin*; and **(D)** *Hymen*. Different letters (a and b) indicate significant differences compared with the control group; *p* < 0.05; Pb = PbCl_2_; relative expression (2^-ΔΔCt^) normalized to CK, CK = control group (50% sucrose solution). L, M, and H represent low, medium, and high lead concentrations, respectively.

Genes involved in gut detoxification and immune defense, namely *GST, PPO, defensin*, and *Hymen*, were also investigated in this study (Xu et al., 2013; Cotter et al., 2019; Liu et al., 2020). Overall, *GST* expression increased significantly with lead concentration in a dose-dependent manner, with the H group showing significantly higher expression than the CK, L, and M groups (*p* < 0.05). However, differences between the L and M groups were not significant (Figure 3A). In the case of *PPO* expression, it decreased slightly in the L group but increased in the M and H groups. At the same time, no significant differences were observed between the low-concentration group (Group L) and the control group, while a significant difference was found between the high-concentration group (Group H) and the medium-concentration group (Group M) (*p* < 0.05) (Figure 3B). Furthermore, the expression of *defensin* was also elevated in all lead-treated groups. Notably, markedly higher expression was observed in the M and H groups compared with CK (*p* < 0.05) as well as between the M/H groups and the L group (Figure 3C; *p* < 0.05). Finally, *Hymen* expression was highest in the H group and significantly greater than in all other groups (Figure 3D) (*p* < 0.05).

### 3.5 Lead alters microbial diversity in bumblebee intestines

**Figure 4:**
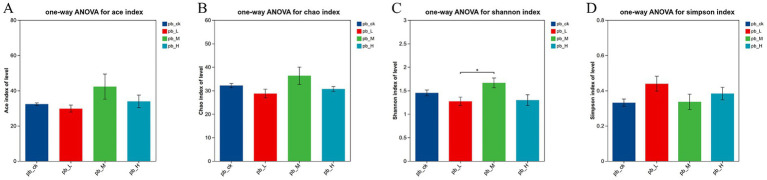
Bar chart showing the *α*-diversity indices of the gut microbiota in bumblebees. The α diversity of the gut microbiota was analyzed after 14 consecutive days of lead exposure. **(A,B)** Illustrate the Chao and ACE indices, indicative of species richness, while the Shannon and Simpson indices **(C,D)**, respectively show species diversity. The box plots depict the median line (central horizontal line), the interquartile range (boxes), and the 95% confidence intervals. *p < 0.05, **p < 0.01, and ***p < 0.001.

The effects of exposure to different lead concentrations on the gut microbiota of bumblebees were studied. The *α* diversity of gut microbes in bumblebees under different lead concentrations was evaluated using the Chao, ACE, Simpson, and Shannon indices (Figure 4). The Chao and ACE indices, which reflect species richness, were higher in the moderate-concentration lead treatment group (group M) than in the control group (CK group) and the low-concentration group (group L). The low-concentration lead treatment group (L group) had the lowest species richness (Figure 4A: ACE index; Figure 4B: Chao index).

The Shannon index was significantly higher in the medium-dose group (group M) than in the low-dose group (group L) (*p < 0.05*), and the Shannon index in the high-dose group (group H) was slightly lower than that in the control group (CK group), the Simpson index was higher than that of the control group (CK group) but lower than that of the low-dose group (L group) (Figure 4C: Shannon index; Figure 4D: Simpson index).

In summary, exposure to moderate lead concentrations resulted in a gut microbiota with high levels of richness and species diversity, but low species dominance, whereas exposure to low lead concentrations significantly reduced richness and greater dominance by a few species. Different levels of lead exposure therefore had distinct effects on regulating the *α*-diversity indices of the gut microbiota.

### 3.6 Altered composition of bumblebee gut microbiota after lead exposure

**Figure 5:**
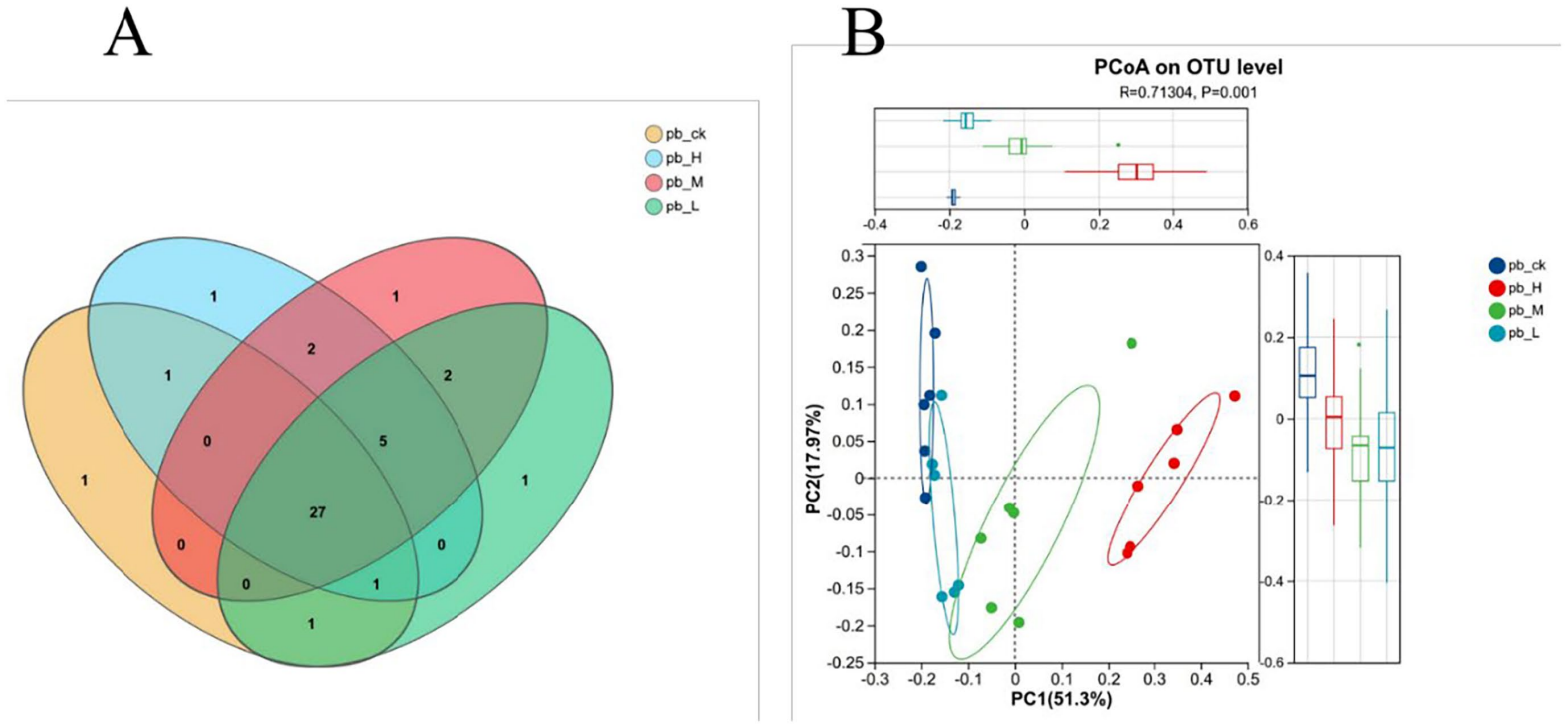
Lead altered the gut microbiota composition of bumblebees. **(A)** Venn diagram showing the distribution of observed OTUs across treatment groups. **(B)** Visualization of the bacterial communities in bumblebee intestines using a PCoA plot using the Bray-Curtis distance.

At a 97% similarity threshold, 32 OTUs were found to be shared among the four experimental groups, with the results presented in a Venn diagram. Thus, despite exposure to three different concentrations of lead, a subset of microbial species remained common across all treatment groups. These coexisting microorganisms likely comprise the core of the gut microbiota in bumblebees. It is noteworthy that each group, including CK, L, M, and H, possesses a unique OTU sequence (Figure 5A), indicating that lead exposure altered the species diversity within the gut environment and introduced certain specific types of microorganisms. This emergence of group-specific OTUs further suggested that the lead treatment led to the enrichment of certain microbial taxa not present in other groups. The principal component analysis (PCoA) results based on the Bray-Curtis distance indicated that lead exposure significantly altered the overall structure of the gut microbiota in bumblebees. The first two principal components, PC1 and PC2, accounted for 51.3 and 18.7%, respectively, of the total variance, with a combined explanatory power of 70.0%. This suggests that these components can effectively capture the differences in microbiota composition between the groups. Statistical tests confirmed the significance of the differences in gut microbiota structure between the groups (R = 0.71304, *p* = 0.001), indicating that lead exposure is a key factor driving the differentiation of gut microbiota composition (Figure 5B). Overall, the distinct spatial separation among groups suggested increasing lead concentration significantly disrupted the native gut microbiota (*p* < 0.05), leading to the formation of distinct microbial community.

### 3.7 Lead concentration-dependent regulation of the gut microbiota and the distribution of dominant bacterial species in bumblebees

**Figure 6:**
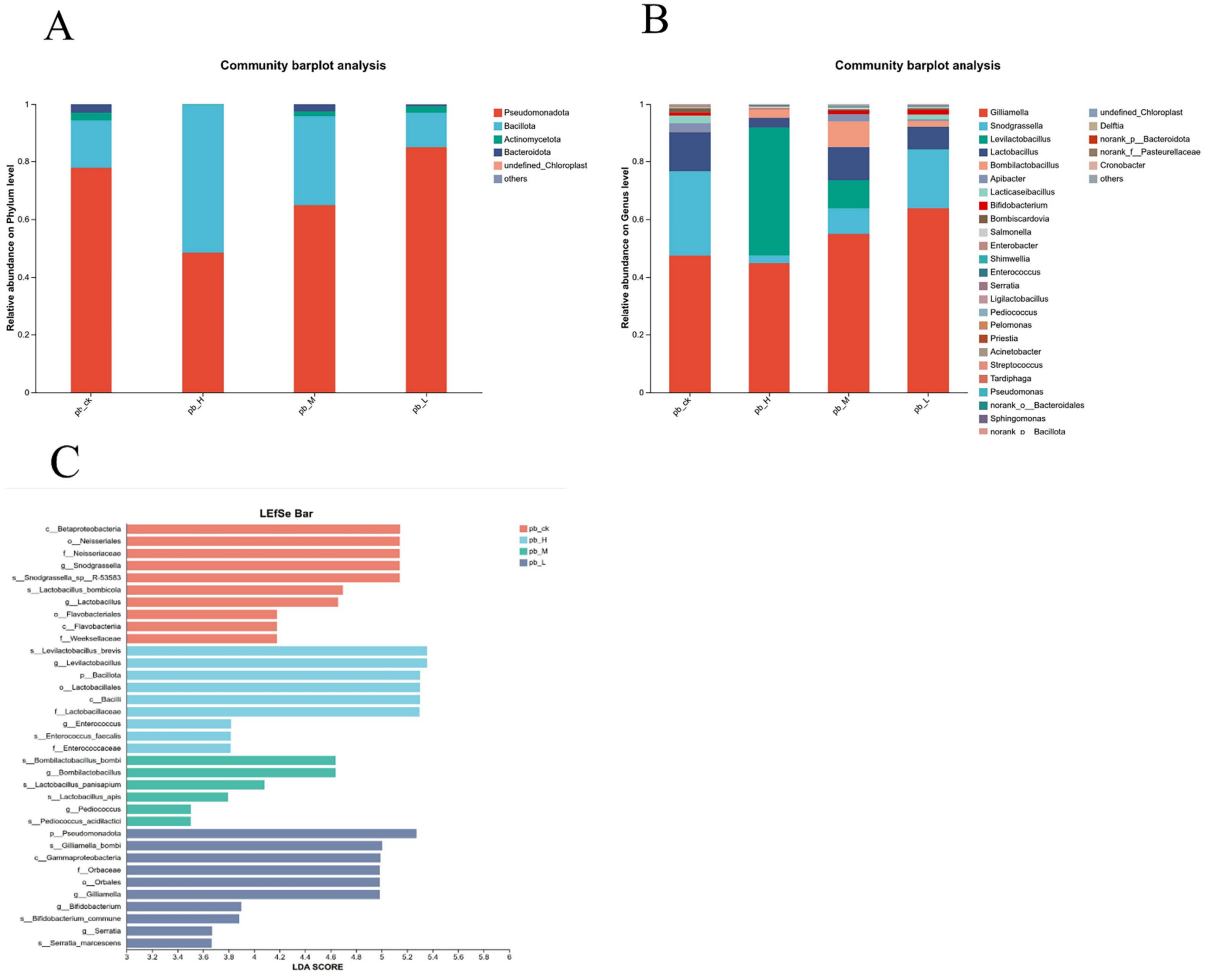
Effects of exposure to different concentration of lead on the composition of the bumblebee gut microbiota. **(A,B)** Column chart of gut microbiota composition (left: phylum level; right: genus level); different colors represent different taxonomic units, columns correspond to treatment groups (CK, control group; L, low lead group; M, medium lead group; H, high lead group); **(C)** Column chart of LDA scores from LEfSe differential analysis (LDA threshold > 2.0, *p* < 0.05).

After exposure of *Bombus terrestris* to different concentrations of lead, significant differences were observed in the composition of its gut microbial communities. At the phylum level (Figure 6A), the core and dominant phyla in the gut microbiota were *Pseudomonadota* and *Bacillota*, which collectively accounted for 70% of the total gut bacterial community. The results showed that the relative abundance of *Pseudomonadota* decreased with the increase in lead concentration, dropping from 25.3% in the control group to 18.5% in the high-lead-concentration group. In contrast, the relative abundance of *Bacillota* increased with rising lead concentration, increasing from 55.2% ± 3.1% in the control group to 68.7% in the high-lead-concentration group. At the genus level, the relative abundance of the beneficial genus *Lactobacillus* decreased significantly with the increase in lead concentration, ultimately dropping to 7.2% in the high-lead group (Figure 6B). Additionally, linear discriminant analysis effect size (LEfSe) was used to identify signature microbial taxa with significant differences among the groups (Figure 6C). In the control (CK) group, the enriched taxa were dominated by core symbiotic bacteria, including *c_Betaproteobacteria*, *g_Neisseria*, *s_Snodgrassella* sp.*_R-55953*, and *s_Lactobacillus bombicola* (LDA > 4.0), indicating that these taxa are the dominant symbiotic bacteria in the gut of *Bombus terrestris*. The low lead-concentration group (L group) was mainly enriched with taxa such as *p_Pseudomonadota*, *g_Gilliamella*, and *s_Bifidobacterium commune*, with LDA scores ranging from 3.0 to 4.0. The changes in the abundance of these taxa represent the early response of the gut microbiota to low lead concentration exposure and reflect mild fluctuations in the intestinal microecosystem. The medium lead-concentration group (M group) had signature taxa including *s_Bombilactobacillus bombi*, *s_Lactobacillus panisapium*, and *g_Enterococcus*, with LDA scores between 2.5 and 3.5. Some species of *Enterococcus* are opportunistic pathogens, which indicated that the symbiotic balance of the gut microbiota was disrupted by harmful bacteria when *B. terrestris* was exposed to moderate lead concentrations. The high lead-concentration group (H group) showed the most significant enrichment of taxa such as *s_Levilactobacillus brevis*, *p_Bacillota*, and *o_Lactobacillales* (LDA > 5.0). These taxa represent the adaptive dominant microbiota under lead concentration stress, with *Levilactobacillus brevis* as the core tolerant species against high lead toxicity.

## 4 Discussion

In recent years, research has demonstrated that lead toxicity can adversely impact the homing ability, foraging behavior, communication and cognitive functions of honeybees (Battisti et al., 2021). In fact, at the molecular level, lead has also been shown to alter the expression of immune and detoxification genes, thus posing a substantial threat to the Chinese honeybee (Shi et al., 2024). Therefore, this study investigated the effects of lead pollution on bumblebees, providing new evidence for understanding the impact of heavy metal contamination on pollinating insects and raising several important questions for future research.

The acute toxicity experiment revealed a median lethal concentration (LC_50_) of 3945.3 mg/L for *B. terrestris*, which was significantly higher than the 345 mg/L reported for honeybees (Di et al., 2016). This indicated a comparatively higher lead tolerance in bumblebees. When exposed to varying concentrations of lead, the expression of genes involved in detoxification and immunity increases in bumblebee, accompanied by remodeling of the gut microbiota in response to different lead concentrations as an adaptation to environmental changes (Xu et al., 2013). Additionally, the physical and anatomical characteristics of bumblebees may confer a certain degree of protection against toxicants. However, despite their greater tolerance, prolonged exposure to lead-contaminated environments significantly reduced the survival and food intake of bumblebees. Indeed, as lead concentrations increased, mortality rose, while feeding behavior declined. These findings were consistent with those of Miguel Corona et al. who reported similar effects of arsenic and selenium exposure in wild bees (Alburaki et al., 2019). The observed reduction in food intake could be attributed to lead-induced damage to gustatory receptors, which impaired the bees’ ability to perceive and respond to food stimuli. Alternatively, the lead might have disrupted normal gut function, impairing digestion and nutrient absorption, thereby reducing food consumption. Such effects not only compromise the growth, development and energy reserves of individual bumblebees but may also jeopardize the survival and reproductive success of entire colonies, which rely on adequate nutritional intake by foraging individuals (Vaudo et al., 2018).

Chronic heavy metal exposure can alter the expression of memory-associated genes in bumblebee. This context, the *DopR1* and *DopR2* genes, which encode dopamine receptors, are crucial for nervous system function (Raza and Su, 2020). In this study, *DopR1* expression increased with rising lead concentrations in a dose-dependent manner, with the H group exhibiting significantly higher expression than the CK group. Similarly, *DopR2* expression was upregulated in the lead-exposed groups, and the highest expression levels were observed in the H group for which expression significantly exceeded that in the CK, L and M groups. Regarding the *NMDA gene*, which plays a key role in synaptic plasticity and is essential for learning and memory in the central nervous system (Wang and Peng, 2016), expression was also markedly increased in all lead-treated groups. These results suggested that lead exposure can alter neural gene expression associated with cognitive functions in bumblebees. This abnormal elevation in signal intensity indicates that lead can damage their nervous systems, which is consistent with the findings observed in western honeybees exposed to varying concentrations of lead, which subsequently affected their learning and memory (Monchanin et al., 2021).

As vital pollinators, bumblebees and honeybees have attracted growing attention in studies in order to address their responses to pesticide and heavy metal exposure (Gong and Diao, 2017). Glutathione S-transferase *GST* and polyphenol oxidase *PPO* are key detoxification and immune-related enzymes involved in neutralizing toxic substances and defending against pathogenic threats in insects (Claudinos et al., 2006). In the current study, *GST* expression was significantly upregulated in the high-dose lead group, indicating that detoxification pathways were activated in response to elevated lead levels. This finding was consistent with the results of Hernandez et al. (2018), who reported that *GST* played a key role in acaricide resistance in ticks. On the other hand, *PPO* expression showed a biphasic response: it was slightly suppressed at low lead concentrations but significantly increased at higher doses. This pattern suggested that low-level lead exposure may be insufficient to trigger immune activation, while higher doses may induce oxidative stress that subsequently stimulate *PPO* expression to enhance immune and antioxidant defenses (Bournoville et al., 2023). Finally, the *Hymen* gene, which encodes an antimicrobial peptide involved in gut immunity, was the most highly expressed in the high-dose lead group. This indicated that elevated lead concentrations may activate gut immune mechanisms in bumblebees to combat lead-induced physiological stress.

In this study, expression of the defensin and Hymen genes was significantly upregulated in the medium-lead group (*p* < 0.05), while the Chao and Shannon indices of the gut microbiota were higher than those in other groups (*p* < 0.05). Moderate immune responses can prevent excessive proliferation of opportunistic pathogens while providing a stable habitat for beneficial bacteria, thereby maintaining the balance of community structure. In contrast, expression of *PPO* genes in the low-lead group showed no significant upregulation (versus the control group). Insufficient immune stress led to the dominance of a few lead-tolerant bacteria (e.g., *Bifidobacterium* spp.), resulting in microbial homogenization (the Shannon index was significantly lower than that in the medium-lead group, *p* < 0.05). In the high-lead group, the Hymen gene (an antimicrobial peptide-encoding gene) was highly expressed. A strong immune response suppressed the most sensitive bacteria, while only the immune-stress-tolerant *Lactobacillus brevis* and *Firmicutes* bacteria were enriched, leading to a decline in community diversity. This finding is consistent with Richardson et al.’s observation (Rothman et al., 2019) that upregulation of *defensin* genes under moderate metal stress can selectively enrich toxin-tolerant bacteria without compromising microbial diversity.

Lead exposure was also found to significantly alter the composition and structure of the gut microbiota in bumblebees. Overall, at a 97% similarity threshold, 32 OTUs were shared across all four experimental groups, with each treatment group also exhibiting unique OTUs. This finding suggested that lead exposure could induce distinct shifts in the gut microbial community. Furthermore, these unique OTUs might have been indicative of microorganisms that responded specifically to lead stress, thus reflecting microbial adaptation or a stress response to lead contamination. Such changes could eventually disrupt the symbiotic relationship between bumblebees and their gut microbiota, with potential consequences for the host’s health. At the species level, lead exposure resulted in a decrease in the relative abundance of *Pseudomonadota* and an increase in *Bacillota*. Previous research has shown that *Pseudomonadota* plays important roles in pathogen suppression and digestion in the insect gut (Jaffar et al., 2022). Hence, a reduction in its abundance may compromise gut immunity and increase disease susceptibility. In contrast, an increase in the relative abundance of *Bacillota* may alter the gut’s metabolic environment (Alberoni et al., 2018), potentially affecting nutrient absorption and utilization. Bumblebees and their gut microbiota form a complex, symbiotic relationship, where in addition to nutrient metabolism, the microbiota also significantly influences the host’s immune responses, physiological functions and behavior. Therefore, lead-induced alterations in the gut microbial composition may negatively affect bumblebee health through multiple mechanisms.

Additionally, LEfSe cladogram analysis provided key insights into the microbial taxa that were significantly affected by lead exposure. These taxa may impact community stability and richness by participating in specific metabolic pathways or competing for ecological niches.

In summary, this study systematically investigated the toxicological effects of lead on bumblebees and elucidated the underlying mechanisms. Notably, long-term exposure to sub-lethal concentrations of lead was found to significantly reduce survival rates and sugar water intake. In addition, lead exposure altered the expression of genes related to learning and memory, detoxification, oxidative stress and immune response, while also affecting gut microbial diversity and composition. Overall, the findings provide new insights into the mechanisms by which heavy metal pollution affects pollinating insects and highlight the importance of future research and conservation strategies.

## 5 Conclusion

Lead is known to exert significant toxic effects on bumblebees. This study demonstrated that prolonged exposure to sub-lethal concentrations of lead reduced bumblebee survival rates and sugar water intake, while also altering the expression of learning and memory-related genes. All of which ultimately altered their feeding behavior. Additionally, lead exposure affected the expression of genes associated with detoxification metabolism and immune responses (*GST*, *PPO*, *defensin* and *Hymen*) and led to notable changes in the richness and composition of the gut microbiota. Collectively, these results deepen current understanding of the physiological and molecular impacts of lead toxicity in bumblebees and offer important insights into the broader ecological risks posed by heavy metal pollutants in the environment. However, the study has several limitations. As these experiments were conducted in a laboratory setting, it was not possible to examine the toxic effects of lead on bumblebees in their natural environment. Furthermore, the study only investigated the toxic effects of lead during 15 days of chronic exposure, and did not consider the long-term impact on bumblebee colonies, for instance, in terms of reproductive success or larval development. The accumulation of lead in bumblebees may also have intergenerational effects, and future research should extend the duration of exposure to assess the consequences at the colony level. In addition, this study did not address potential interactions between lead and other environmental stressors, such as pesticides, habitat loss, or climate change. Pollinating insects in their natural environment are often subjected to multiple stressors simultaneously.

## Data Availability

The data presented herein are raw 16S rRNA gene sequencing data, which have been deposited in NCBI under the accession number PRJNA1470110.
